# Iridociliary cysts do not impact on posterior phakic intraocular lens implantation for high myopia correction: A prospective cohort study in 1569 eyes

**DOI:** 10.1371/journal.pone.0196460

**Published:** 2018-04-26

**Authors:** Xi Zhang, Xun Chen, Xiaoying Wang, Xingtao Zhou

**Affiliations:** 1 Department of Ophthalmology, Eye and ENT Hospital of Fudan University, Shanghai, PR China; 2 Department of Ophthalmology, Myopia Key Laboratory of the Health Ministry, Shanghai, PR China; Boston University School of Medicine, UNITED STATES

## Abstract

**Purpose:**

To investigate the clinical characters and effect of iridociliary cysts among patients who had undergone posterior phakic intraocular lens implantation.

**Methods:**

A total 1569 eyes of 866 high myopia patients, who underwent phakic intraocular lens implantation from 1 September 2011 to 31 August 2016, was included in this prospective cohort study. These patients were followed up for more than 12 months postoperatively.

**Results:**

During the study period, 218 eyes (14%) of 154 patients were diagnosed with iridociliary cysts by ultrasound biomicroscopy. There were 74.3% patients with unilateral cysts and the cysts tended to have occurred in patients ages 20 to 30 years old (22%). The location of the cysts varied with temporal position as the most (50%) and superior position as the least (9.6%). After 1 week postoperative follow-up, there were no significant differences in clinical outcomes between the cysts group and no cysts group, but the proportion of 20/20 or better uncorrected visual acuity (UCVA), best corrected visual acuity (BCVA), and the 2 types of vector astigmatism. Intraocular pressure (IOP), refraction, and vault remained stable at different time points of the follow-up period, while vault of cysts patients tend to be lower than patients without cysts at more than 12 months.

**Conclusions:**

Iridociliary cysts were more common than estimated and it had no impact on Phakic Intraocular Lens implantation because there was no clue to show significantly difference on postoperative clinical outcomes between the patients with and without iridociliary cysts in this study.

## Introduction

In December 2005, the Implantable Collamer Lens (Visian ICL; STAAR Surgical, Nidau, Switzerland), a posterior phakic intraocular lens, was approved for commercial use in the treatment of myopia -3.00D to -20.00D. Then, the Toric Implantable Collmer lens (TICL) was developed to correct -3.00 to -6.00D astigmatism. Currently, the ICL/TICL is widely used for high myopia on a global basis, because this new lens has a greater safety record and has proven to be more effective than laser corneal surgery.[1–4]

Since ICL/TICL was introduced in our hospital, we have been made aware of an increase in the number of iridociliary cysts cases. Iridociliary cysts, the majority of which were located at the iris-ciliary body junction or the top of ciliary processes, were benign cyst lesions.[5] They are rarely found by slit-lamp examination, gonioscopy, or optical coherence tomography (OCT).[6, 7] The large iridociliary cysts can cause anterior chamber angle closure and increase the risk of secondary glaucoma.[8–10] For ICL/TICL implantation, many surgeons take more concern into the cysts impacting on the ideal location and vault of implantable lens in a postoperative short period. And in the long term, whether the ICL/TICL implantable stimulates the iridociliary cysts growth or changes the aqueous humor generation is also noted by many researchers. In traceable study results,[7, 11, 12] the incidence of iridociliary cysts ranged from 4.9% (1157 patients) to 54.3% (116 patients); there has been no consensus and the clinical outcome of only one patient case, who had undergone treatment with the Toric Implantable Collamer Lens, has been reported.

Thus, could Posterior Phakic Intraocular Lens Implantation be performed among patients with iridociliary body cyst and how were the postoperative long-term safety and stability compared with the patients without cysts? These were the primary questions investigated during this study.

## Materials and methods

### Patients

866 patients (1569 eyes) who had undergone implantation with ICL / Toric ICL (STARR Surgical) for the correction of myopia astigmatism between 2010 and 2016 were involved in this study. Informed consent was provided by the participants and the principles outlined in the Declaration of Helsinki were followed in this study. All patients included in this study had to have met these criteria: spectacle and/or contact lens intolerance; stable refraction for at least 1 year before preoperative examination; an endothelial cell density (ECD) ≥2000 cell/mm^2^; anterior chamber depth ≥2.8 mm; and no history of cataract, glaucoma, uveitis, uncontrolled diabetes, collagen vascular disease, or previous intraocular surgery. This work is granted permission by the Ethics Committee of Eye and ENT Hospital, Fudan University.

### Main measurement parameters

In this study, the main measurement parameters were: uncorrected visual acuity (UCVA); best corrected visual acuity (BCVA); manifest and cycloplegic refraction; corneal keratometry; intraocular pressure (IOP); iridociliary cysts’ number, size, location by ultrasound biomicroscopy (UBM) preoperative and postoperative. Besides, postoperative vault and adverse events were important parameters for evaluation. All examinations were taken in the operative eyes.

### Statistical methods

The following statistical analyses were used to compare visual outcomes of the 2 types of high myopia patients after implantation. SPSS statistics software package version 18.0 for Windows (SPSS, Chicago, IL, USA) was used for statistical analysis. The Fisher exact test was performed on dichotomous variables (BCVA or UCVA, 20/20, and 20/40 or better, predictability of ±0.50 or ±1.00D). Mann–Whitney U tests were performed to explore statistical differences for refractive index, efficacy index, safety index, intraocular pressure, and vault among different patient subgroups. p<0.05 was considered significant in all cases.

## Results

### Patients population

This study involved 866 patients (1569 eyes) who underwent ICL/TICL (version 4) and prospected the short-term/long-term clinical outcomes. The mean age of the subjects was 29.30±7.74 years old (range: 18 to 55 years old) and the manifest refraction spherical equivalent (MRSE) was -15.41 ±4.58 D. Among the 866 patients (1569 eyes), 154 patients (218 eyes) had iridociliary cysts. Table 1 provides a comparison of demographics and preoperative sphere and cylinder for the cysts group and no cysts group. We noticed that patients with iridociliary cysts were significantly younger, with 2.2 years difference in average age and with less myopia and astigmatism.

**Table 1:** Population and preoperative refractive comparison of the cysts group and no cysts group.

	Without cysts	With cysts	P value
Gender(%female)	413/712(58%)	103/154 (67%)	0.042**
Age (years old, mean ±SD)	29.70±7.96	27.51±6.38	0.007**
Sphere (D; mean ±SD)	-14.67±4.46	-13.86±4.83	0.009**
Cylinder (D; mean ±SD)	-1.87±1.28	-1.48±1.19	0.001**

*: p<0.05;

**: p<0.01.

### Postoperative follow-up

**Fig 1:**
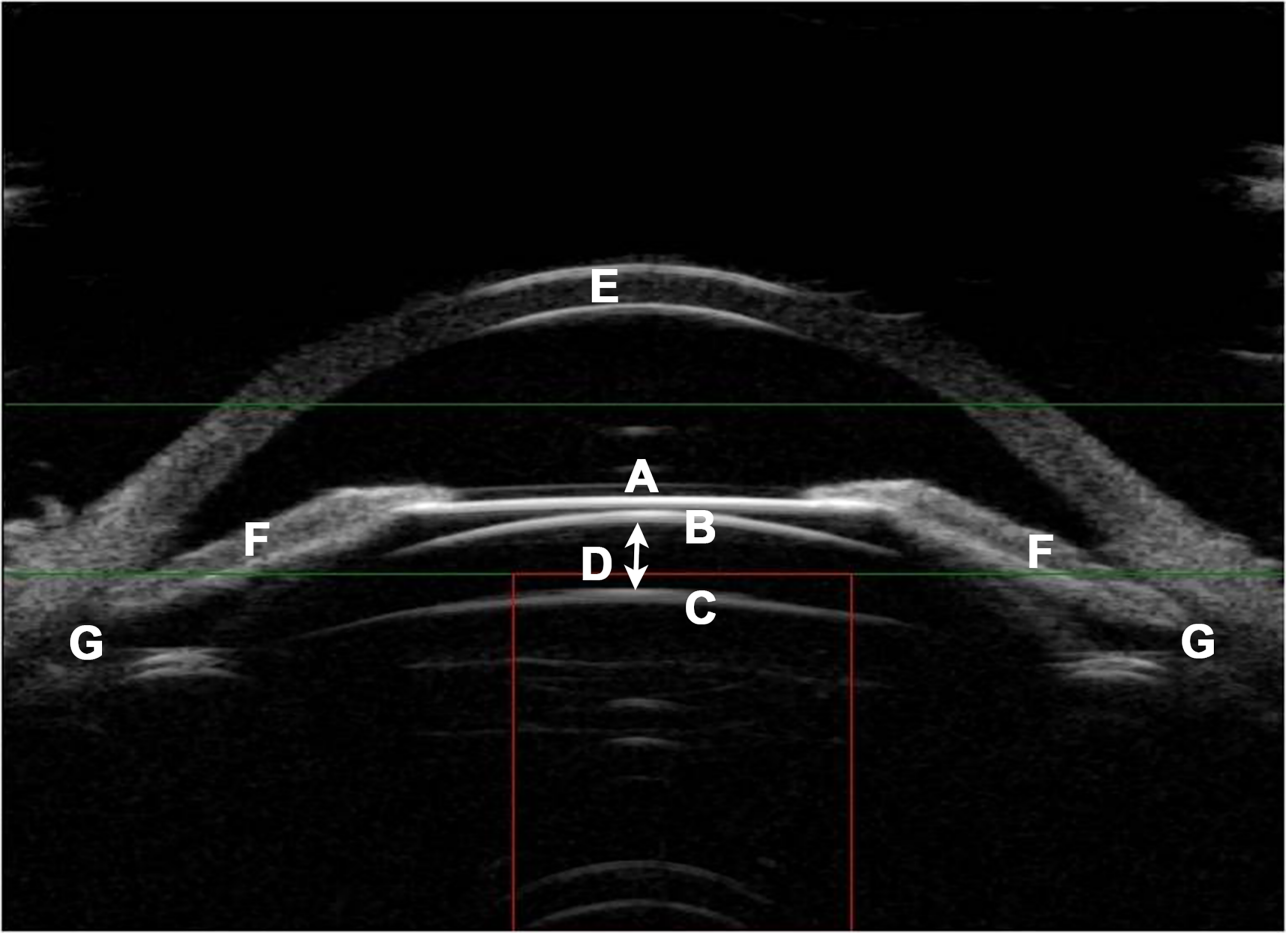
Image of ultrasound biomicroscopy after phakic intraocular lens implantation. A: front surface of Implantable Collamer Lens; B: back surface of Implantable Collamer Lens; C: front surface of crystalline lens; D: vault; E: corneal; F: nasal and temporal iris; G: nasal and temporal ciliary sulcus.

We evaluated 866 patients (1569 eyes) at the following time points: 1 day after surgery, 1 week after surgery with a clinical analysis including UCVA, BCVA (only at 1 week), refraction (only at 1 week), intraocular pressure (IOP), and vault (only at 1 week; the distance between the front surface of crystalline lens and the back surface of Implantable Collamer Lens, Fig 1). We followed up on174 patients (289 eyes) for more than 12 months among the 866 patients and evaluated the clinical examination (UCVA, BCVA, refraction, IOP, vault measurement) at 1 month, 3 months, 6 months and≥12 months (the last visit). The mean follow time of last visit in two groups was 16.96 +/- 7.75 months and 19.18 +/- 9.04 months, respectively.

### Clinical characters of iridociliary cysts

As mentioned above, 218 (14%) of 1569 eyes were diagnosed with iridociliary cysts. Patients aged between 20 and 30 years old had the highest incidence rate (105/474; 22%) of iridociliary cysts, compared with the incidence rate in patients under 20 years old (16%), in patients ranging in age from 31 to 40 years old (15%), and in patients over 40 years old (5%).

**Fig 2:**
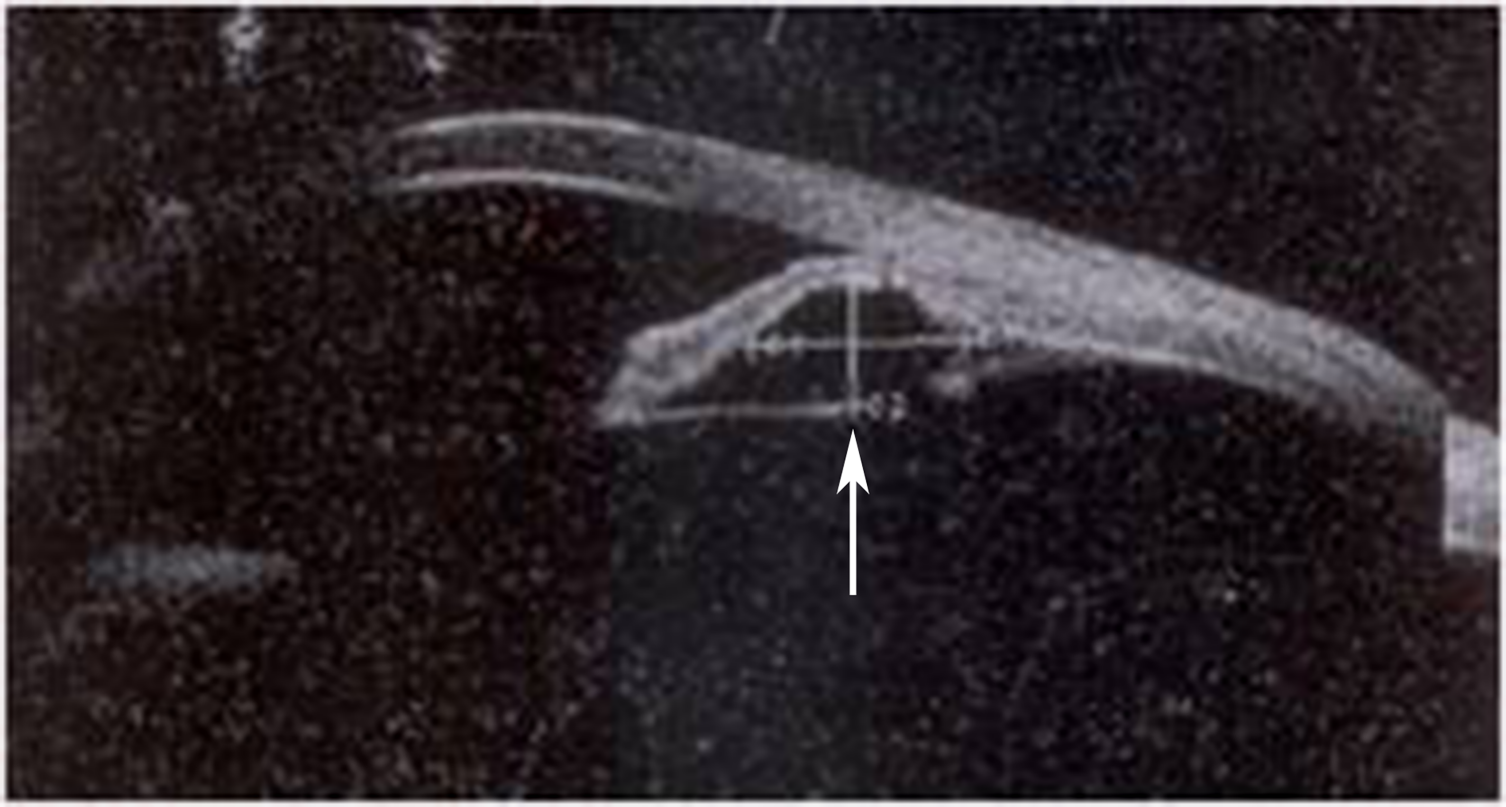
Image of the patients who had a huge iridociliary body cysts at the ciliary sulcus. The arrow shows the iridociliary body cysts.

The 218 eyes of 154 patients with iridociliary cysts had different characters of distribution (unilateral or bilateral), numbers (single or multiple), location, and size. Among the 154 patients, 138 patients underwent binocular ICL/TICL implantation. There were 75 (54.3%) of 138 patients with unilateral cysts, compared with 63 (45.7%) of 138 patients (63/138) with bilateral cysts. The proportion of eyes with a single cyst was 50% (109/218); this equaled the number of eyes having multiple cysts. The iridociliary cysts had 8 locations in the eyes, including nasal (14%), superior nasal (10%), inferior nasal (16.5%), temporal (50%), superior temporal (36%), inferior temporal (41%), superior (9.6%) and inferior (29%). All cysts were less than 1 mm except in one eye of a female patient; this one cyst was quite large with a diameter >2 mm (Fig 2). In the 2 eyes (1.9%) of one patient, cysts were detected almost completely around the 360° ciliary body.

### Short-term clinical outcomes

**Fig 3:**
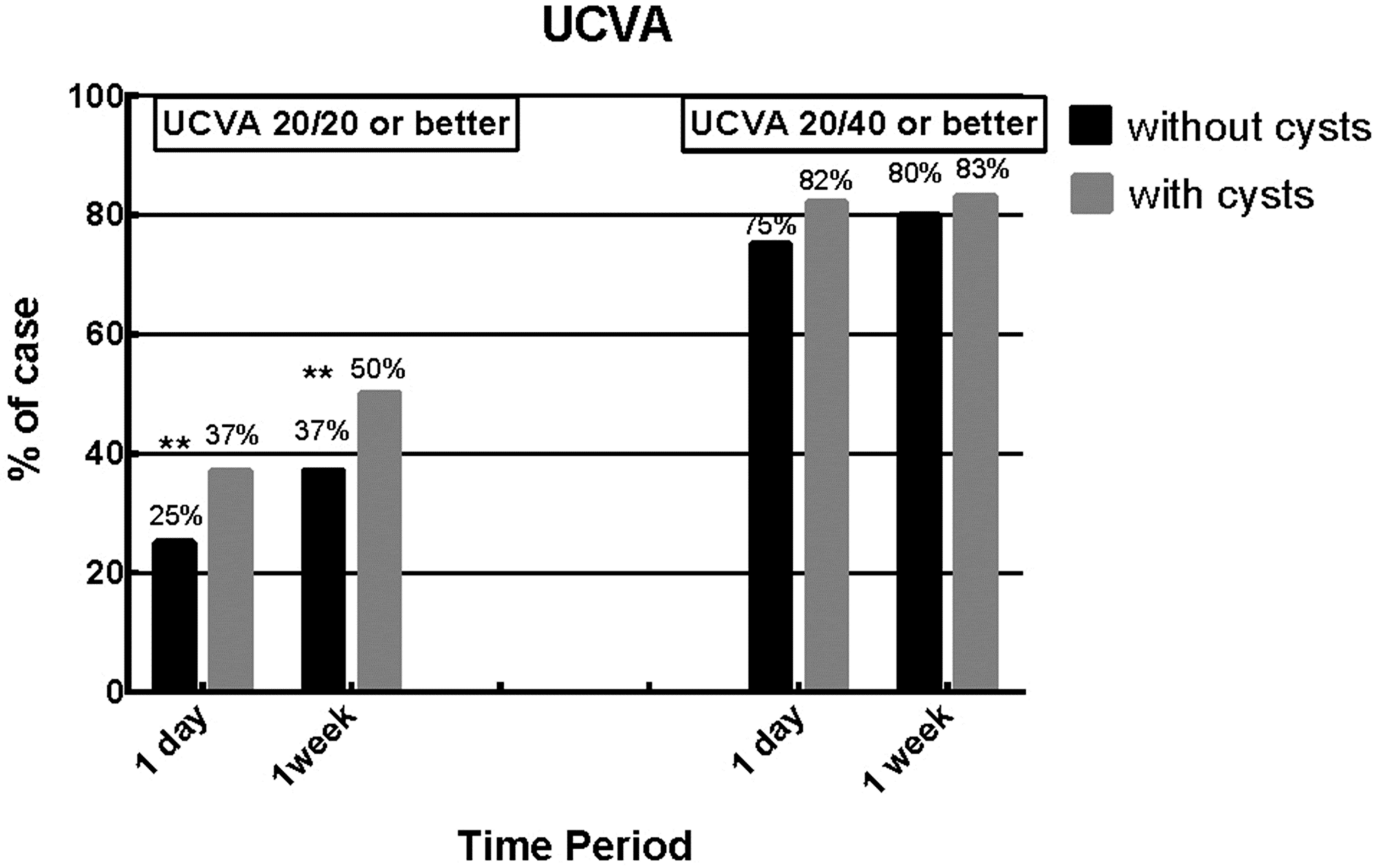
Iridociliary body cysts group versus patients without iridociliary body cysts uncorrected visual acuity (UCVA) 20/20 or better and 20/40 or better at postoperative one day and one week. (*: P<0.05; **: P<0.01; cysts group: 1 day, n = 207; 1 week, n = 205; no cysts group: 1 day, n = 1216; 1 week, n = 1068).

**Fig 4:**
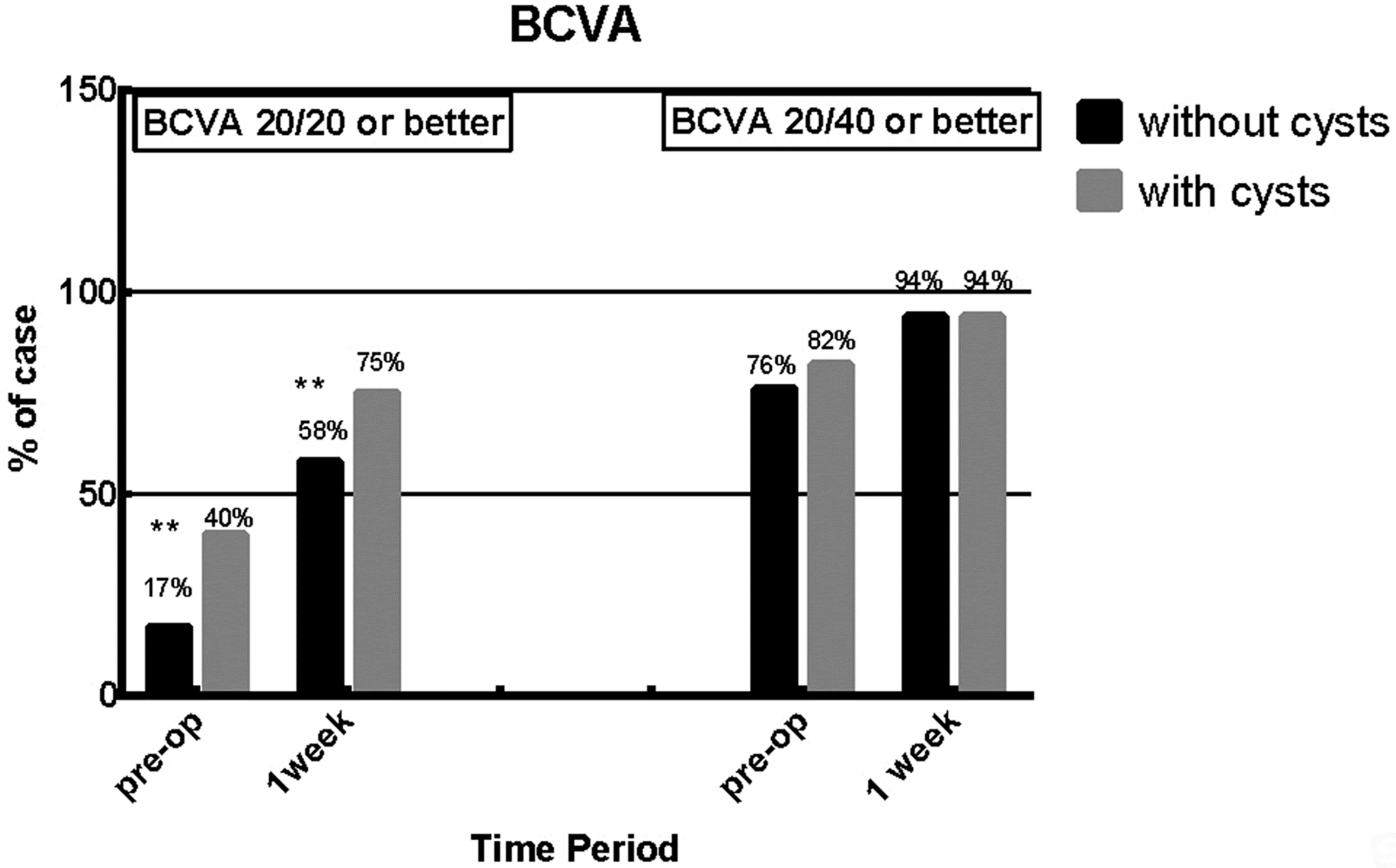
Iridocilirary body cysts group versus patients without iridocilirary body cysts best corrected visual acuity (BCVA) 20/20 or better and 20/40 or better at pre-operate and postoperative one week. (**: P<0.01; cysts group: pre-op, n = 218; 1 week, n = 205; no cysts group: pre-op, n = 1351; 1 week, n = 1068).

All eyes had an uncorrected visual acuity (UCVA) of 20/200 or worse prior to surgery. After the implantation of ICL/TICL, the UCVA of 2 groups improved dramatically. In both groups, the proportions of cases achieving the 20/40 or better UCVA (Fig 3, p>0.05) and 20/40 or better BCVA in both groups (Fig 4, p>0.05) had no significant statistical differences in each follow-up visit. However, the proportion of cases with 20/20 or better UCVA in group with cysts was significantly higher than the other group at one day and 1 week postoperatively (Fig 3, p<0.01; p<0.001). Besides, at 1 week and preoperative, the proportions of cases achieving the 20/20 or better BCVA (Fig 4, p<0.01; p<0.001) showed the same significantly difference with 20/20 or better UCVA.

The efficacy index (mean postoperative UCVA/mean preoperative BCVA) and safety index (mean postoperative BCVA/mean preoperative BCVA) in cysts group were 1.11+/-0.34 and 1.35+/-0.45, respectively, during the 1week follow-up visit period. The efficacy index and safety index were 1.16+/-0.55 and 1.48+/-1.03, respectively in the second group. Both the safety index and efficacy index showed no significant differences between the 2 groups (p>0.05).

**Fig 5:**
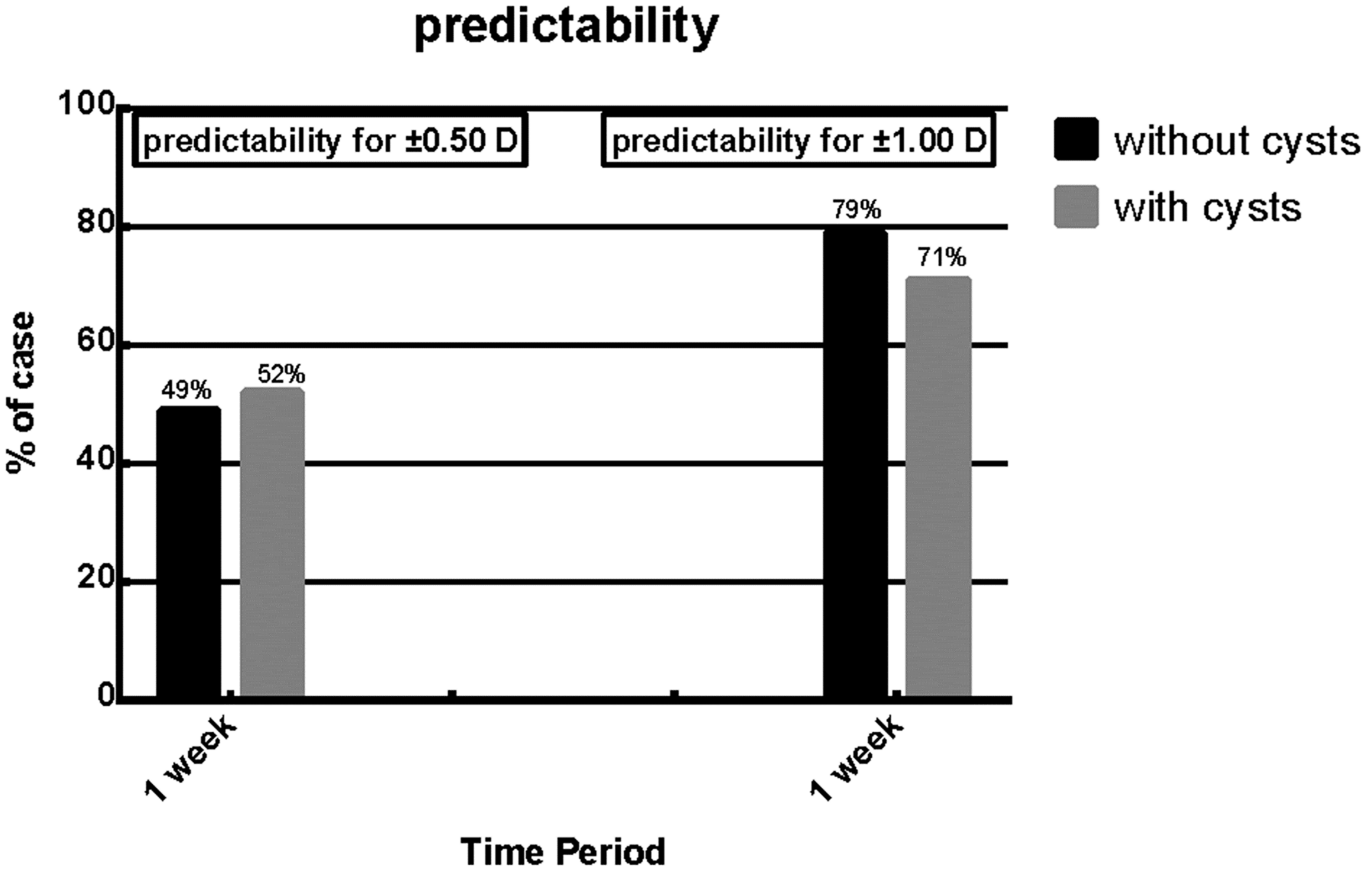
Predictability for ±0.50 D and ±1.00D of iridocilirary body cysts group versus patients without iridociliary body cysts during the 1week postoperative period.

The mean spherical equivalent, J45 astigmatism (axes at 45° and 135°) and J0 astigmatism (axes at 180° and 90°) have no statistical difference at 1week after the implantation surgery (Table 2). Compared to the proportion of predictability (attempted versus achieved correction) for ±0.50 D and ±1.00 D between the 2 groups, there was no different at 1 week visit period. (Fig 5; p>0.05).

**Table 2:** Comparison of the refractive outcomes in the two groups at preoperative and at the one week postoperative period.

Variable		No cysts group	Cysts group	P value
Mean spherical equivalent Refraction (MSE, mean ±SD)	Pre-op	-15.21±4.58	-13.70±6.35	0.001**
	1week	-1.06±1.48	-0.95±1.60	0.410
J0 (mean ±SD)	Pre-op	-0.75±0.65	-0.13±0.62	0.338
	1week	-0.05±0.39	-0.07±0.38	0.635
J45 (mean ±SD)	Pre-op	0.02±0.77	0.06±0.70	0.519
	1week	0.04±0.49	0.01±0.47	0.389

**: p<0.01. UCVA: uncorrected visual acuity; BCVA: best corrected visual acuity; J0: Jackson cross-cylinder, axes at 180° and 90°; J45: Jackson cross-cylinder, axes at 45° and 135°.

### Short-term intraocular pressure and vault

Table 3 shows the comparison of intraocular pressure (IOP) and vault between the 2 groups at different follow-up visits. There was no statistical difference in the cysts group and the no cysts group.

**Table 3:** Short-term comparison of intraocular pressure (IOP) and vault in the two groups after ICL implantation.

	IOP			vault		
	Without cysts	With cysts	P value	Without cysts	With cysts	P value
1 day	14.66±3.66	14.80±4.02	0.677	--	--	--
1 week	15.86±3.95	16.01±4.58	0.686	538.05±210.22	496.71±176.67	0.103

### Long–term clinical outcomes and changes of iridociliary cysts

#### Clinical outcomes

**Fig 6:**
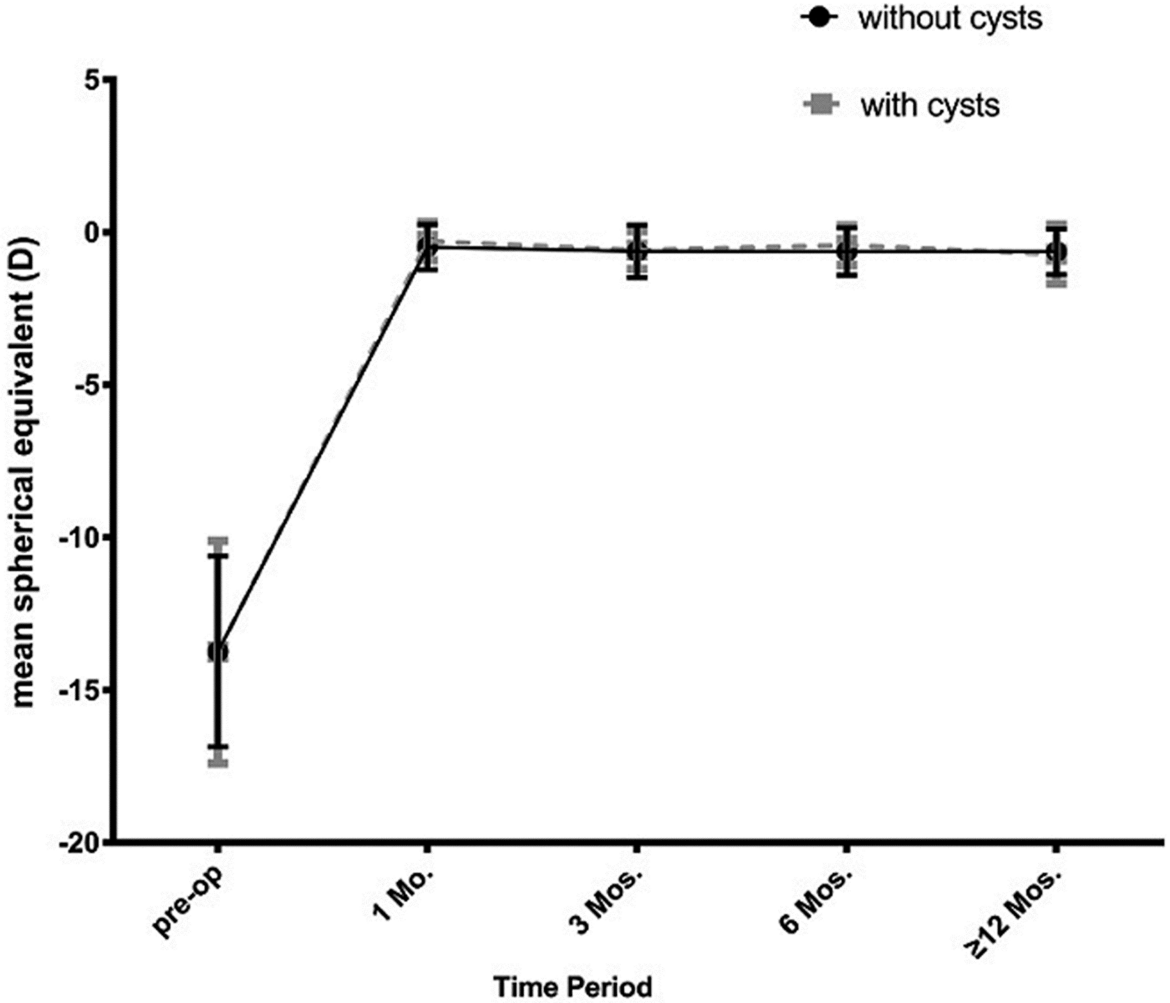
Change of mean spherical equivalent in the 2 groups at pre-operate and different postoperative visit periods.

**Fig 7:**
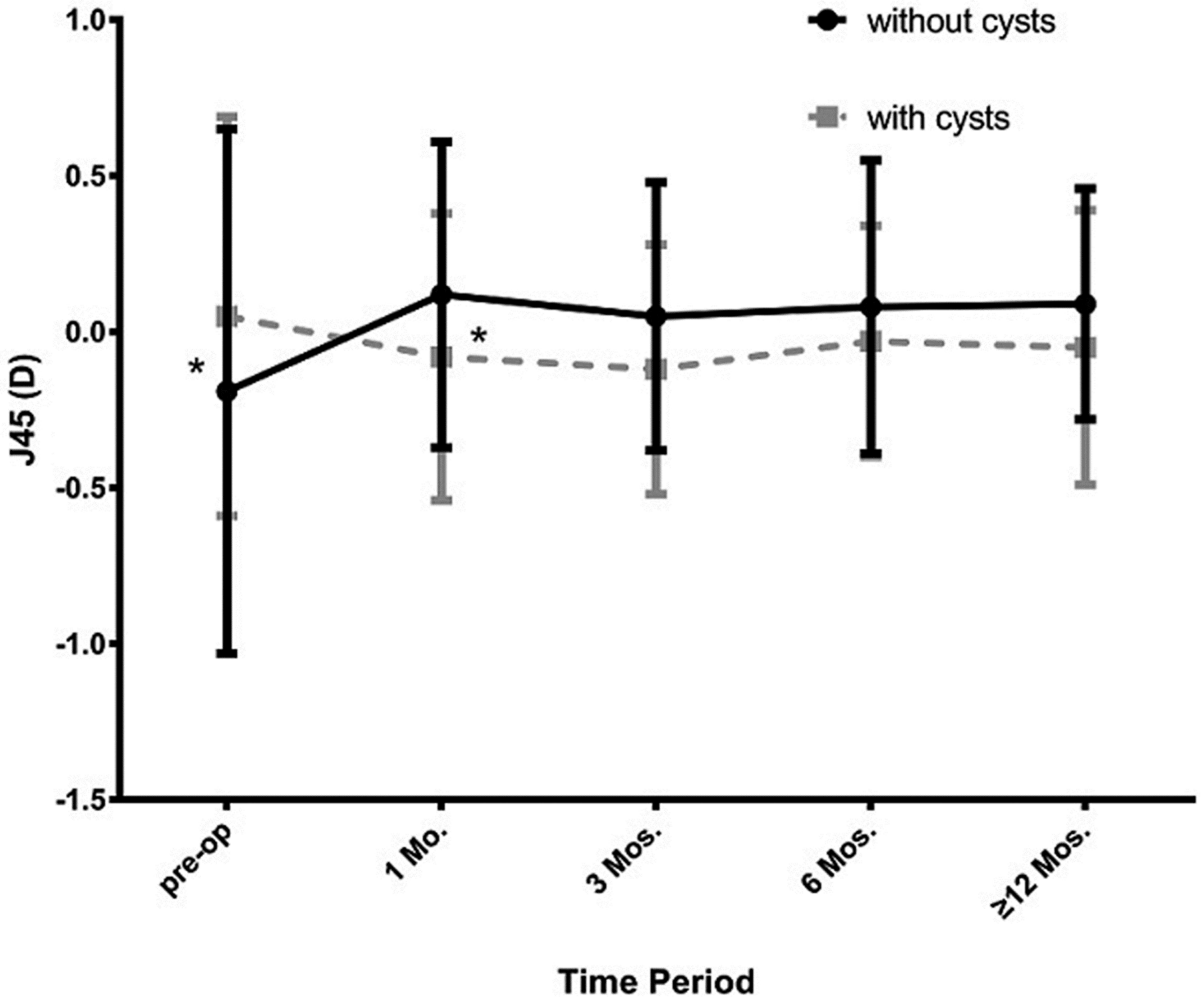
Change of J45 astigmatism of the 2 groups at pre-operation and at different postoperative visit periods.

**Fig 8:**
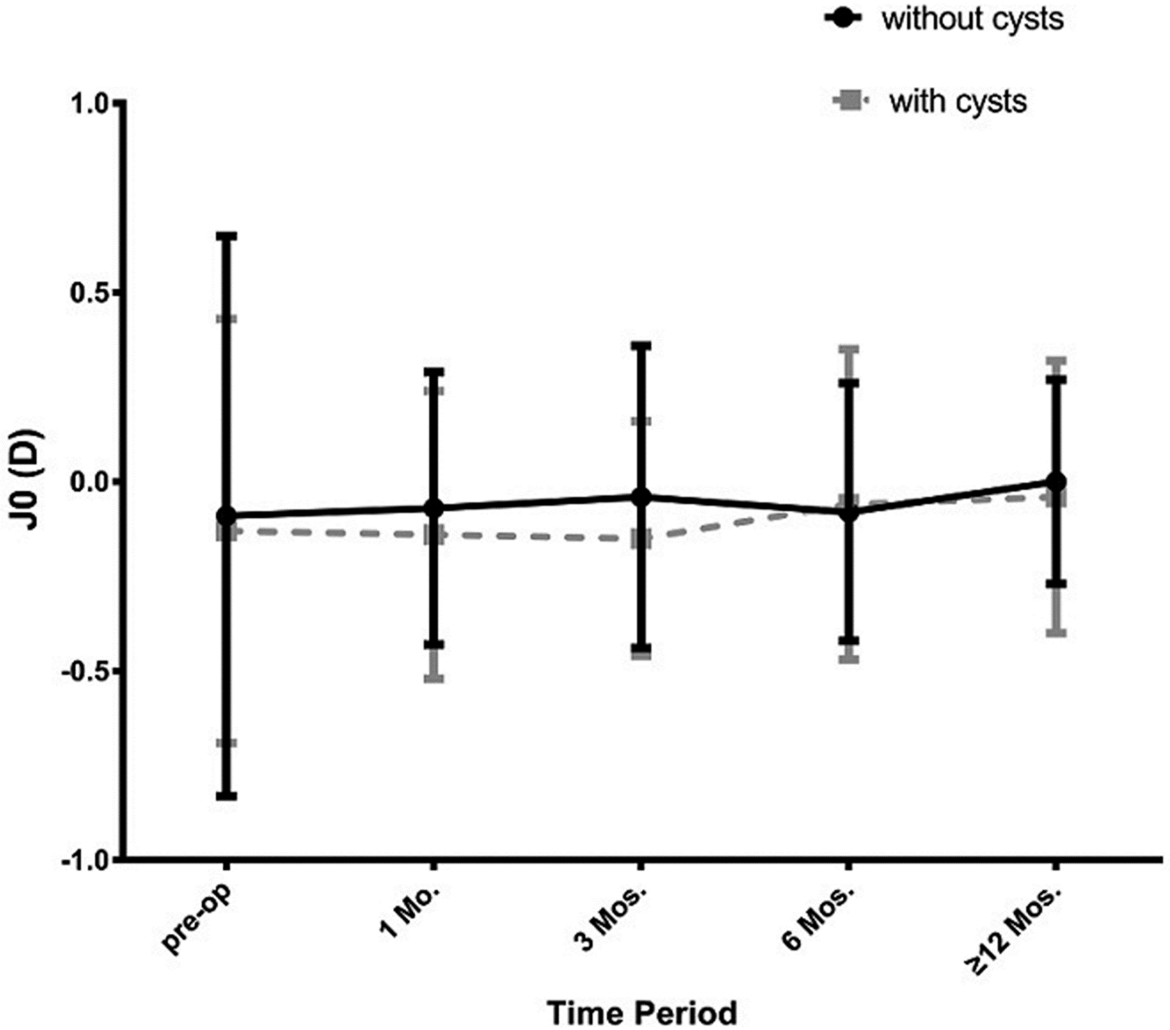
Chang of J0 astigmatism in the 2 groups at pre-operation and different postoperative visit periods. *: p<0.05.

A total of 289 eyes (174 patients) were followed for more than 12 months. Among them, there were 108 eyes of 74 patients that had iridociliary cysts and 181 eyes having no cysts. During the last visit period, the efficacy index (1.09±0.36 vs 1.08±0.35; P>0.05) and safety index (1.30±0.36 VS 1.23±0.39; p>0.05) of the 2 groups showed no statistically significant differences. The mean spherical equivalent in the cysts group was statistically lower than the no cysts group, only at the one month visit (Fig 6; -0.29±0.62D vs -0.48±0.74D, p<0.05). With statistically different preoperative J45 astigmatism of the 2 groups (Fig 7; -0.19±0.84D vs 0.05±0.64D, p<0.05), we found that J45astigmatism in the cysts group was lower than the no cyst group at 1 month (-0.08±0.46D vs 0.12±0.49 D, p<0.05). Both the mean spherical equivalent and the 2 types of victor astigmatism of 2 groups, remained stable at all visits (Figs 7 and 8).

**Fig 9:**
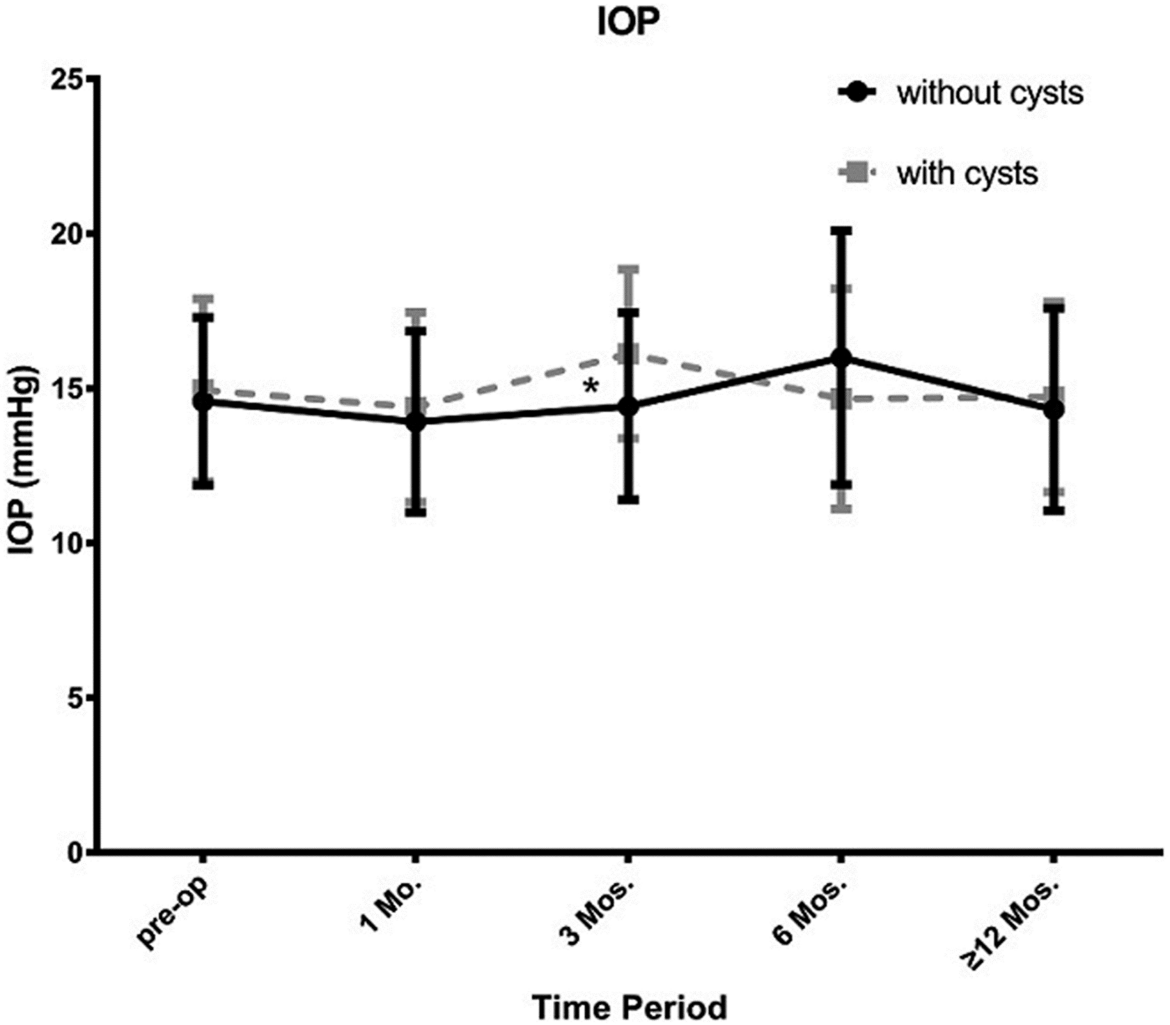
Change in intraocular pressure in the 2 groups at pre-operation and different postoperative visit periods. *: p<0.05.

**Fig 10:**
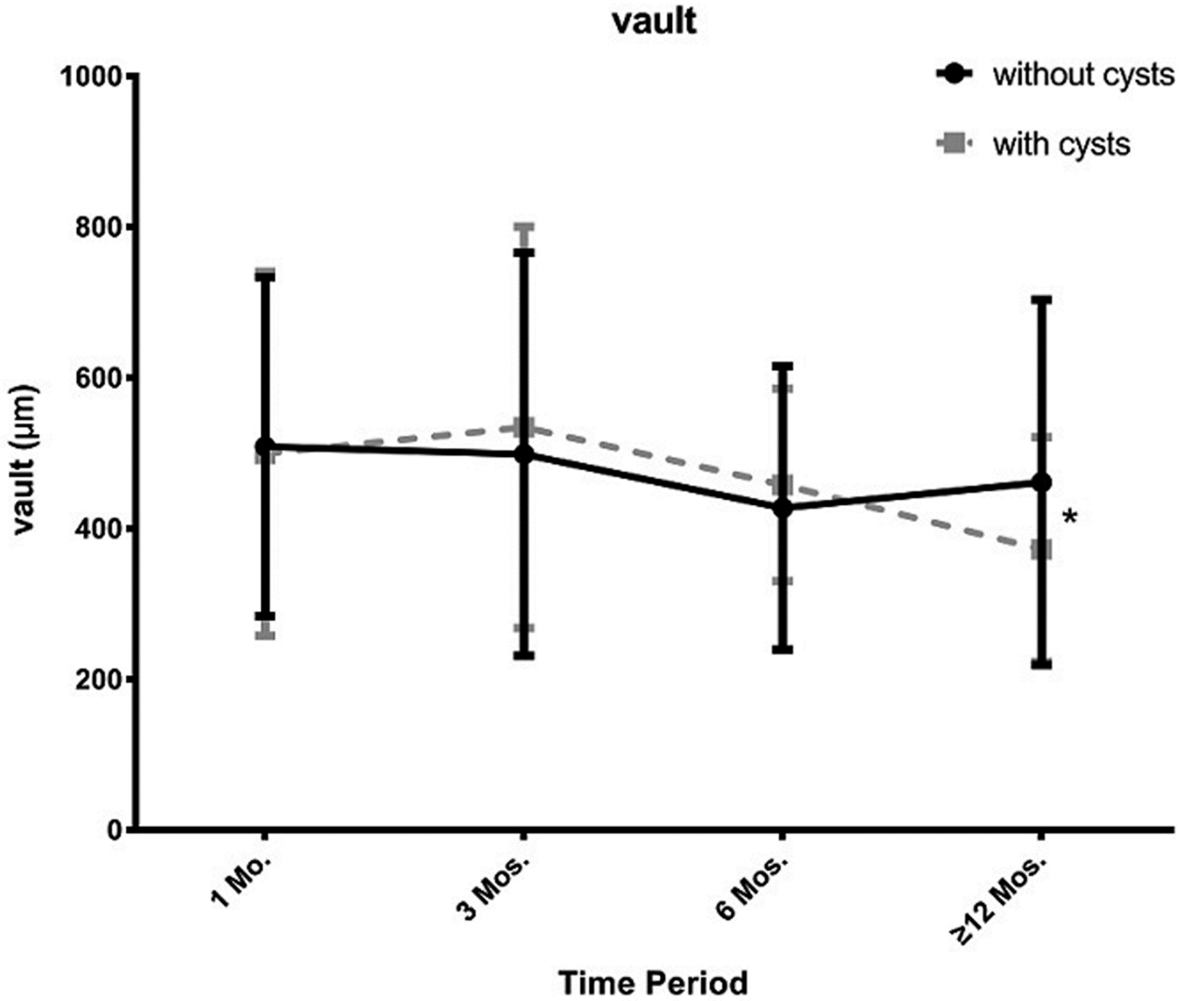
Change in vault in the 2 groups at different postoperative visit periods. *: p<0.05.

**Fig 11:**
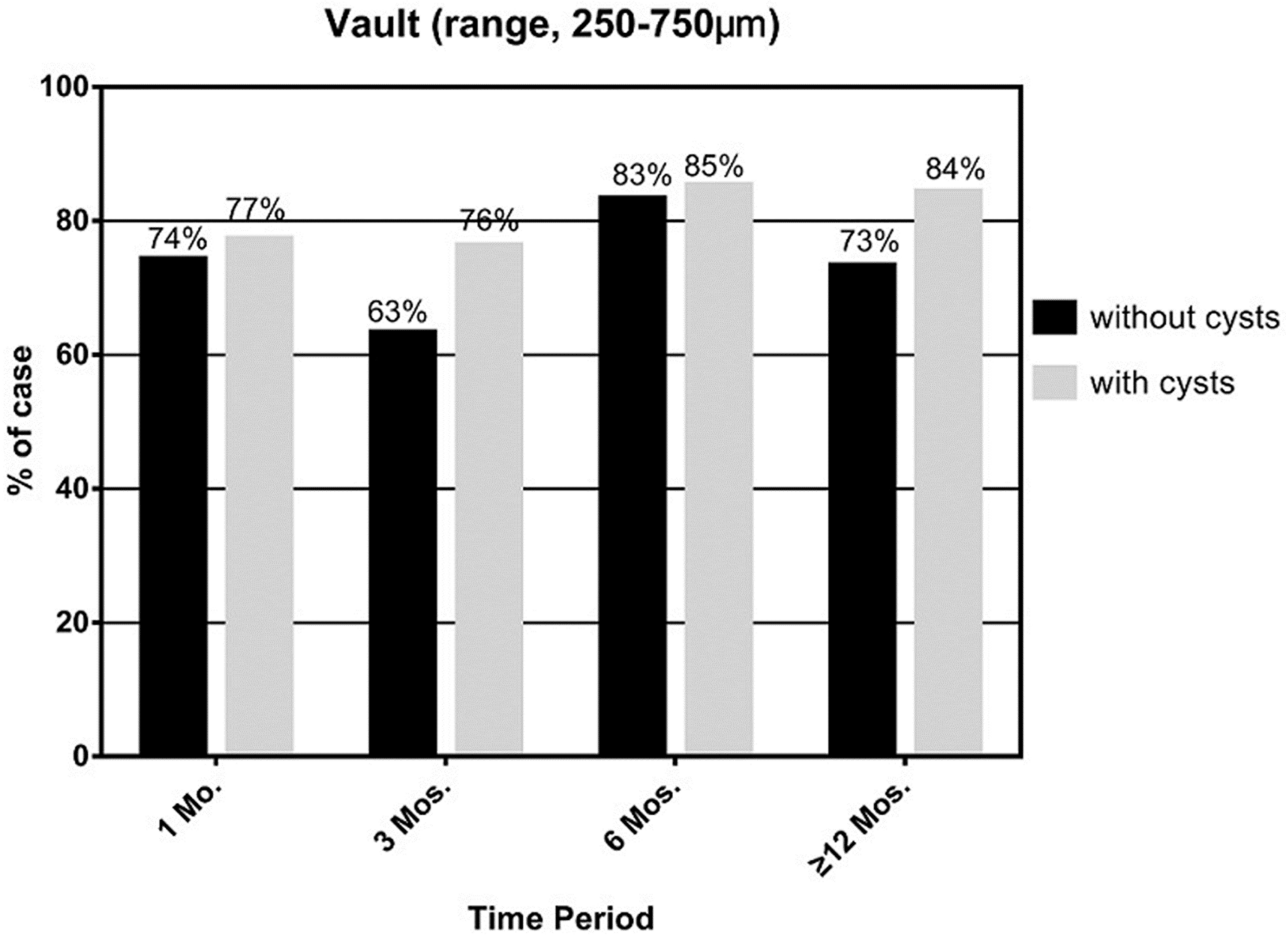
The ideal vault (250–750 μm) proportion of the 2 groups at different postoperative visit periods.

At the 3-month postoperative visit period, IOP in cysts groups was significantly higher than the no cysts group (16.12±2.73mmHg vs14.43±3.02 mmHg, p<0.05), while the vault of the cysts group was lower than the other group at the last visit period (398.52±170.36 μm vs 502.73±254.94 μm, p<0.05). Figs 9 and 10 show trends in IOP and vault at all follow-up visits. We noticed that in both groups, intraocular pressure kept stability and had no significant difference at every follow-up visit while the vault in cysts group showed more tended to decrease than the no cysts group. However, the proportion of ideal vault (ranges from 250–750 μm) in the 2groups was no significant difference at all visit time period (Fig 11; p>0.05).

#### Changes of iridociliary cysts

A total of 33eyes with iridociliary cysts, who had undergone implanted ICL/TICL were examined by ultrasound biomicroscopy in October, 2017. The mean follow time was 35.4 +/- 15.75 months (ranged from 7 to 72months). The mean cyst diameter was 0.94+/- 0.41mm (ranged from 0.5 to 1.87mm). We mainly observed whether the cysts changed in quantity and position between preoperative and the last visit after surgery. Most eyes (16 eyes, 48.5%) had no changes, while the inferior iridociliary body cyst diameter in only 1eye became larger than preoperative. Among 9 eyes with cysts, 8eyes showed the preoperative existed cysts disappeared completely and 1 eye exhibited that only the inferior cysts had disappeared. There was no statistically significant difference in age (P>0.05) in each group. Table 4 shows the changes in 33 eyes with iridociliary cysts at the last visit.

**Table 4:** Changes in 33 eyes with cysts at the last visit.

Changes	Eyes (%)	Age (year, mean +/- SD)
No changes	16 (48.5%)	31.27+/-6.37
Size enlarged	1 (3.0%)	31
Disappear	9 (27.3%)	30.44+/-5.83
Decrease number	2 (6.1%)	31.00+/-5.30
Increase number	5 (16.2%)	28.75+/-3.30

#### Adverse events

In the process of long-term follow-up, no eye in our study had any intraoperative complications (Table 5). In the group without cysts, 5 (2.8%) of 181 eyes had symptoms of glare after implantation surgery. Meanwhile, there were 3 eyes (2.1%) that had the same symptoms in the other group. We noted the dark pupil diameters of all eyes were larger than 7.0 mm, and 4 eyes dark had pupil diameters that were larger than 7.5mm. Symptoms of glare persisted only for a short period.

**Table 5:** Adverse events among the two groups.

Adverse Events	NO. Eyes	Result
Glare		
No cysts group	5(2.8%)	Resolved after 12 months
Cysts group	3(2.1%)	Resolved after 12 months
Macular hemorrhage		
No cysts group	1(0.5%)	No significant decrease in BCVA
Cysts group	0	
Retinal Detachment		
No cysts group	0	
Cysts group	1(0.7%)	Given fundus operation
ICL/TICL adjustment		
No cysts group	4(2.2%)	Vault decreased/corrected astigmatism
Cysts group	0	

For one patient, there were the need to adjust the ICL position due to the high vault (>1100μm) at the one week follow-up visit, and in one patient the need to adjust the TICL axial because of the increased astigmatism at the 12 months visit time period in the group without cysts. After the adjustment, the vault decreased to less than 1000μm; the astigmatism of the other patient was corrected.

Fundus disease was detected in both groups after surgery. At 20 months post initial surgery, one eye in the no cysts group experienced macular hemorrhage; this did not cause the significant decrease of BCVA, and one eye in cysts group occurred retinal detachment 25 months after surgery, which lead to a loss of 7 lines in BCVA compared to preoperative.

## Discussion

Since the advent of ultrasound microscopy in early 1990s, we started to become more familiar with iridociliary cysts. These cysts were far more common than we had estimated. One case having solitary temporal iris cysts had been reported to followed up 15 months after TICL implantation and there was no impact on final visual outcome or TICL position.[11] In our study, we identified the characters of iridociliary cysts and evaluated the cysts effects on clinical outcomes of ICL/TICL implantation at different postoperative visit periods.

Among the 1569 eyes of 866 patients in present study, we found the prevalent of iridociliary cysts was 14%, which was closer to previous 4.9% of 1157 patients. Similar to a previous study,[13] these cysts were more commonly diagnosed in women (67%) and at approximately 20 to 30 years of age (22%). Most cysts located at the temporal, superior temporal, inferior temporal.

ICL/TICL was implanted to the posterior chamber and the implantable lens’ haptics were positioned into the sulcus. Since iridociliary cysts most commonly appear at the iridosciliary body junction or the top of ciliary processes, the haptics may be affected by the ciliary cysts. If the haptics are not placed into the appropriate position, the corrective effect and lens’ stability will be affected.[14] Meanwhile, at one day after ICL/TICL implantation, clinical outcomes including UCVA, BCVA, IOP, and vault may not reflect the real outcomes because of the small pupil and a greater tendency to be influence by various factors. While at postoperative one week period, the visual outcomes, refractive outcomes, IOP and Vault could keep stable in common.[15,16] Therefore in this study, we followed 1569 eyes of 866 patients at 1 day and 1 week and 289eyes of 176 patients, who were followed over 12months after phakic intraocular lens implantation to evaluate the effect of iridociliary cysts at different visit periods.

After the one week follow-up period, the MSE, 2 types of vector astigmatism (J0, J45) of two groups were corrected adequately in the 2 groups. Based on the analyses of the results, we noticed that except for the proportion of UCVA 20/20 or better and the proportion of BCVA 20/20 or better in cysts group exhibiting higher than no cysts group which might due to the statistical preoperative significant difference in BCVA 20/20 or better (p<0.05). Predictability for ±0.50 D and ±1.00D and refractive outcomes showed no significant difference, which indicated that this current study is a fairly representative of the outcomes reported with approved implantable collamer lens clinical trials.

At the process of over 12 months follow-up, we noticed both groups’ mean spherical equivalent, J0 and J45 astigmatism remained stable at all postoperative visit periods. Furthermore, we compared more than 1year postoperative iridociliary cysts changes in size, number, and position, with preoperative, and we noticed that the cysts changed in half of the eyes. Among the changing cysts, most eyes (27.3%) had cysts disappearing and we speculated that because the haptics of ICL might break the cysts intraoperative. Thus far, we could not determine the relationship between phakic IOL implantation and iridociliary cysts changes due to the small sample. In the future, we could enlarge the sample size and further analyze in order to acquire the more substantial evidence.

A posterior chamber phakic intraocular lens, with the haptics on the ciliary sulcus, is designed to be implanted in the posterior chamber behind the iris and in front of the anterior capsule of the crystalline lens.[17] The haptics and anterior vault are to minimize contact with the crystalline lens. Achieving an ideal vault is one of the factors to as a certain postoperative safety. Gonvers at al [18] reported that poorly-sized vault will lead to some complications such as to cataracts, angle-closure glaucoma, and pigment dispersion syndrome. The vault could be affected by various factors such as ICL/TICL sizes, age, ACD, and haptic position.[19] In our study, The vault in the cysts group decreased more significantly than in the no cysts group which we supposed to be due to the difference in sample size. However, the proportion of patients having an ideal vault show no statistical difference at all postoperative time periods.

Some study reported that the large iridociliary cysts would cause anterior chamber angle closure and increase the risk of secondary glaucoma. We followed the only one eye of a patient in our study who has a ciliary body cyst diameter >2mm and found that the spherical equivalent ranged from 0 D to +0.50 D, the IOP and vault were 17.4mmHg and 460μm at 12 months follow up visit. More cases with huge iridociliary cysts could be evaluated in the future to summarize the character of the >2mm diameter iridociliary cysts. Mc Whae et al [20] described 73 patients with multiple bilateral iridociliary cysts using ultrasound microscopy and found that 9.1% cases occurred glaucoma requiring treatment and 9.1% cases had a narrow angle. In addition, the cyst-related glaucoma occurred in patients that had multiple areas of angle compromise due to large cysts. This study did not investigate the safety and effectiveness between multiple and single iridociliary cysts patients after ICL/TICL implantation. In future, we could perform a prospective study to further explore this area.

In conclusion, iridociliary cysts are mostly located on the temporal (including temporal, superior temporal, inferior temporal) of the eyes and the diameters of cysts usually less than 1mm. There was no impact on the postoperative 1 week and over 12 months clinical outcomes in cysts group and they did not increase the intra-operative and postoperative risk. Furthermore, most iridociliary cysts have no changes in size, number after long term postoperative follow-up visit. The results in our study indicate that ICL/TICL implantation could performed in patients with iridociliary cysts.

## Supporting information

S1 File Minimal data. Partial data of some parameters examined before the operation. IOP: intraocular pressure; WTW: distance of white to white; K: keratometry; ACD: anterior chamber depth.
(XLSX)
